# Draft Genome Sequence of FT9, a Novel *Bacillus cereus* Strain Isolated from a Brazilian Thermal Spring

**DOI:** 10.1128/genomeA.01027-14

**Published:** 2014-10-09

**Authors:** Tainá Raiol, Marlene Teixeira De-Souza, João Victor A. Oliveira, Helena Schubert da Incarnação Lima Silva, Juliana Capella Orem, Danilo Andrade Cavalcante, Nalvo F. Almeida, Guilherme P. Telles, João Carlos Setubal, Marcelo M. Brigido, Fernando A. G. Torres, Peter S. Stadler, Maria Emília M. T. Walter, Lídia M. P. Moraes

**Affiliations:** a Department of Cellular Biology, University of Brasilia, Brasília, Brazil; b Department of Computer Science, University of Brasilia, Brasília, Brazil; c Department of Computing and Statistics, Federal University of Mato Grosso do Sul, Campo Grande, Mato Grosso do Sul, Brazil; d Institute of Computing, UNICAMP, Campinas, São Paulo, Brazil; e Department of Biochemistry, University of São Paulo, São Paulo, Brazil; f Department of Computer Science and Interdisciplinary Center for Bioinformatics, University of Leipzig, Leipzig, Germany; g The Santa Fe Institute, Santa Fe, New Mexico, USA

## Abstract

A *Bacillus cereus* strain, FT9, isolated from a hot spring in the midwest region of Brazil, had its entire genome sequenced.

## GENOME ANNOUNCEMENT

*Bacillus cereus sensu lato* is a group comprising ubiquitous aerobic spore-forming bacteria, which includes *B. cereus sensu stricto*, an opportunistic pathogen involved in poisoning food and systemic and local infections, the anthrax pathogen *B. anthracis*, and four other species (1, 2). The members of this group historically have been subdivided into pathogens and environmental strains of medical, industrial, and ecological relevance. Originally distinguished only on the basis of their phenotypic differences, the evolutionary history of this important bacterial group is being uncovered by large-scale comparative genome sequence analyses, showing that this classification is not commensurate with phylogenetics.

A very broad thermal interval for growth temperature in the *B. cereus* group strains has been reported, ranging from 4°C to 50°C (3). However, only very few strains of the *B. cereus* group are able to grow at temperatures of >48°C. In fact, this ability seems to be restricted to the genetically distant strain *B. cereus* NVH391-98 (4), isolated from a severe food poisoning outbreak, which caused three fatal cases (5), and to a few nonpathogenic thermotolerant strains (4).

Here, we present the draft genome sequence of *B. cereus* strain FT9, isolated from a hot spring located in the midwest region of Brazil. Interestingly, strain FT9 is able to grow at least at 52 ±1°C and thus can be considered a new thermotolerant strain presenting potential heat resistance gene products.

The complete genome of FT9 was sequenced by a whole-genome shotgun approach using 454 FLX/Roche. A total of 584,619 reads were obtained, with an average length of 428 bp. The circular chromosome is 5,223,665 bp long and was assembled using MIRA (6), using the *B. cereus* ATCC 10987 sequence as a reference genome. The overall DNA G+C content is 35.5%, similar to that of other *B. cereus* group species. Open reading frames (ORFs) were predicted using GRC (7) and annotated using BLAST (8) and bacterial proteins from the PATRIC database (9). Out of 5,743 ORFs, 5,191 (90.4%) were annotated by their similarities as coding for known protein functions, 108 (1.9%) were considered conserved hypothetical proteins, and 552 (9.6%) were considered hypothetical genes, without any significant hits. The rRNAs and tRNAs were identified using RNAmmer (10) and tRNAscan-SE (11), respectively. From these analyses, 92 tRNAs and 12 *rrn* operons, comprising 5S, 16S, and 23S rRNA genes, were detected in the chromosome.

### Nucleotide sequence accession numbers.

The results from this whole-genome shotgun project have been deposited with DDBJ/EMBL/GenBank under the accession no. CP008712. The version described in this paper is the first version, CP008712.1.
